# High-Accuracy Off-Grid Sparse Bayesian Learning with Reliability-Guided Inference for Direction-of-Arrival Estimation

**DOI:** 10.3390/s26144459

**Published:** 2026-07-14

**Authors:** Wenchao He, Haoran Wang, Hongxi Zhao, Yiran Shi

**Affiliations:** 1 School of Mechanical and Electrical Engineering, Changchun Humanities and Sciences College, Changchun 130117, China; hewc23@mails.jlu.edu.cn; 2 College of Communication Engineering, Jilin University, Changchun 130012, China; hrwang23@mails.jlu.edu.cn (H.W.); hxzhao24@mails.jlu.edu.cn (H.Z.)

**Keywords:** direction-of-arrival estimation, off-grid sparse Bayesian learning, basis mismatch, multiple-measurement-vector model, reliability-guided inference

## Abstract

Off-grid direction-of-arrival (DOA) estimation based on sparse Bayesian learning (SBL) can alleviate angular discretization mismatch, but its practical performance may be affected by unreliable posterior relevance statistics, sensitivity of effective error precision learning, and unstable offset correction. This paper proposes a reliability-guided stabilized off-grid SBL method for multisnapshot DOA estimation. The method is developed within the standard first-order multiple-measurement-vector Bayesian model and introduces three stabilization modules. First, a confidence-guided MAP-type shrinkage relevance update is introduced to suppress weak and non-dominant posterior components through reliability-controlled non-expansive shrinkage. Second, a posterior-concentration-guided damped noise update is introduced to stabilize scalar effective error precision learning when the sparse support is uncertain. Third, a trust-region cubic-regularized Newton refinement is formulated to obtain bounded active-support off-grid corrections from the posterior expected reconstruction error. Simulation results under off-grid deviation, varying SNRs, varying snapshot numbers, different source separations, and random-angle scenarios show that the proposed method achieves competitive and stable estimation performance compared with representative classical and sparse Bayesian baselines.

## 1. Introduction

Direction-of-arrival (DOA) estimation is a fundamental task in array signal processing, with applications in radar, sonar, wireless communications, and intelligent sensing. Since practical observations are often affected by noise, finite snapshots, and model mismatch, DOA estimators are expected to provide both accurate and stable localization results [1,2,3,4].

Classical DOA estimation methods include beamforming-based, subspace-based, and maximum-likelihood-based approaches. Capon proposed the minimum-variance distortionless response beamformer to improve spatial resolution by suppressing undesired interference [5]. MUSIC exploits the orthogonality between signal and noise subspaces for high-resolution estimation [6], while ESPRIT uses rotational invariance to avoid exhaustive spectral searching under suitable array structures [7]. Maximum-likelihood estimators can achieve high statistical efficiency, but usually require nonlinear multidimensional optimization [8]. Although these methods are well established, their performance depends strongly on reliable sample statistics and accurate array models. In low-SNR or limited-snapshot scenarios, the sample covariance may deviate from its ideal value, and the separation between the signal and noise subspaces becomes less reliable. These factors can lead to degraded resolution, missed sources, or unstable estimation results.

Sparse reconstruction provides another route for high-resolution DOA estimation. By discretizing the angular domain into an overcomplete dictionary, DOA estimation can be formulated as sparse spatial spectrum recovery. Malioutov et al. introduced this sparse reconstruction perspective for source localization [9]. Orthogonal matching pursuit (OMP) is a representative greedy sparse recovery method with low computational cost [10]. However, because OMP selects atoms sequentially, its estimation result can be sensitive to noise and early selection errors. More importantly, most fixed-grid sparse reconstruction methods suffer from basis mismatch when the true DOAs do not lie exactly on the predefined grid. This mismatch may spread the source energy over neighboring grid atoms and reduce the accuracy of the recovered sparse spectrum, especially when a coarse grid is used to control computational complexity.

To improve the stability of sparse recovery, sparse Bayesian learning (SBL) introduces a probabilistic inference framework for estimating sparse coefficients and relevance hyperparameters. Based on automatic relevance determination, SBL estimates the posterior distribution of sparse coefficients and learns relevance hyperparameters within a Bayesian framework [11,12]. Bayesian compressive sensing further showed that sparse signals and noise uncertainty can be jointly inferred from limited measurements [13]. Compared with greedy sparse methods, SBL exploits posterior mean, posterior covariance, and hyperparameter learning, making it attractive for multisnapshot DOA estimation with shared row sparsity. In the multiple-measurement-vector (MMV) setting, the common sparse support across snapshots can be used to improve estimation stability. However, conventional grid-based SBL still relies on a fixed angular dictionary and therefore remains affected by basis mismatch in off-grid scenarios.

To alleviate the basis-mismatch problem, Yang et al. proposed off-grid sparse Bayesian inference (OGSBI), in which first-order perturbation variables are introduced to characterize the deviations between true directions and their nearby grid points [14]. Dai et al. developed RootSBL, which improves continuous-angle refinement and computational efficiency through root-based off-grid estimation [15]. Subsequent studies have improved off-grid SBL from several representative aspects, including adaptive noise modeling, sparse prior design, continuous-angle refinement, and efficient Bayesian inference. Wen et al. introduced a variational sparse Bayesian framework for off-grid DOA estimation under nonuniform noise, showing that off-grid perturbations and noise uncertainty can be jointly inferred [16]. Yang and Yang incorporated hierarchical synthesis Lasso priors to enhance sparsity promotion and support discrimination for off-grid signals [17]. Shen et al. proposed an improved RootSBL method by further refining the root-based update strategy of the original RootSBL framework [18]. More recently, Li et al. developed an off-grid SBL estimator with three-stage hierarchical Laplace priors, and Tong et al. proposed an off-grid fast variational Bayesian inference method to reduce computational burden [19,20]. Deep-unfolding SBL networks have also been investigated to combine model-driven Bayesian inference with data-driven learning [21].

Although off-grid SBL alleviates angular basis mismatch by introducing continuous offset variables, first-order off-grid inference may still be affected by unreliable relevance learning, sensitive effective error precision updating, and unstable offset refinement. These issues can weaken support concentration and lead to less stable off-grid correction, especially under finite snapshots and noisy observations. Therefore, this paper focuses on stabilizing these sensitive inference steps within the first-order off-grid MMV-SBL framework.

To address these issues, this paper proposes a reliability-guided stabilized off-grid SBL method for multisnapshot DOA estimation. Within a first-order MMV Bayesian framework, CGBS-MAP, PCG-DNU, and TR-CRN are integrated to improve support concentration, stabilize effective error precision learning, and provide bounded off-grid refinement. The main contributions of this paper are summarized as follows:

1. An empirical-Bayes-inspired confidence-guided MAP-type shrinkage relevance update is proposed. By combining posterior confidence and local dominance, the update suppresses weak and non-dominant components. It improves sparse relevance concentration without using a hard support threshold.
2. A posterior-concentration-guided damped noise update is introduced. The residual-based effective error precision update is relaxed according to the current relevance concentration, which stabilizes precision learning during uncertain support formation while preserving the residual-based fixed point.
3. A trust-region cubic-regularized Newton refinement is developed for active-support off-grid correction. By imposing cubic damping and trust-region constraints, the refinement provides bounded offset updates and reduces overly aggressive correction within the local first-order approximation range.

The remainder of this paper is organized as follows. Section 2 introduces the signal model and problem formulation, Section 3 presents the proposed method, Section 4 reports the simulation results, and Section 5 concludes the paper.

## 2. Signal Model

### 2.1. Array Observation and Bayesian Sparse Model

Consider *K* far-field narrowband sources impinging on an *M*-element array from directions ${\left\{\theta_{k}\right\}}_{k=1}^{K}$. Let $x\left(l\right)\in C^{M}$ denote the received snapshot at time index *l*, and let *L* denote the number of snapshots. The array observation model is

(1) x(l)=A(θ)s(l)+n(l),l=1,2,…,L

where $A\left(\theta \right)=\left[a\left(\theta_{1}\right),a\left(\theta_{2}\right),\ldots ,a\left(\theta_{K}\right)\right]\in C^{M\times K}$ is the steering matrix, $s\left(l\right)\in C^{K}$ is the source vector, and $n\left(l\right)\in C^{M}$ denotes additive noise. Stacking all snapshots gives

(2) X=[x(1),x(2),…,x(L)]=A(θ)S+N

where $X\in C^{M\times L}$, $S\in C^{K\times L}$, and $N\in C^{M\times L}$.

For a linear array with sensor positions normalized by wavelength, the steering vector is written as

(3) $$a\left(\theta \right)={\left[e^{-j2\pi p_{1}\;\sin\;\theta },e^{-j2\pi p_{2}\;\sin\;\theta },\ldots ,e^{-j2\pi p_{M}\;\sin\;\theta }\right]}^{T}$$

where $p_{m}=d_{m}/\lambda$ is the normalized position of the *m*th sensor, with $d_{m}$ measured from the reference sensor. For a half-wavelength ULA, $p_{m}=\left(m-1\right)/2$.

After discretizing the angular search interval, the sparse source coefficients over the candidate grid are represented by a row-sparse matrix $W\in C^{N\times L}$. For each snapshot, the sparse coefficient vector is assigned the prior

(4) $$w\left(l\right)∼\mathrm{CN}\;\left(0,\mathrm{diag}(\alpha )\right)$$

where $\alpha ={\left[\alpha_{1},\alpha_{2},\ldots ,\alpha_{N}\right]}^{T}$ is a variance-type relevance hyperparameter vector. A larger $\alpha_{n}$ indicates a more active candidate direction, while a smaller $\alpha_{n}$ suppresses the corresponding grid component.

The residual after sparse grid modeling contains both observation noise and residual modeling error. It is denoted as an effective model error and modeled as

(5) $$e\left(l\right)∼\mathrm{CN}\left(0,\alpha_{0}^{-1}I_{M}\right)$$

where $\alpha_{0}$ is the scalar precision of the effective Gaussian error.

### 2.2. First-Order Off-Grid Sparse Model

The angular search interval is discretized into a grid ${\left\{{\tilde{\theta }}_{n}\right\}}_{n=1}^{N}$. Since true DOAs generally do not exactly coincide with predefined grid points, fixed-grid sparse modeling suffers from basis mismatch.

To compensate for this mismatch, a first-order off-grid approximation is introduced. For a direction around ${\tilde{\theta }}_{n}$, the steering vector is approximated as

(6) $$a\left(\theta \right)\approx a\left({\tilde{\theta }}_{n}\right)+b\left({\tilde{\theta }}_{n}\right)\beta_{n}$$

where, for an active grid point close to a true DOA, $\beta_{n}=\theta -{\tilde{\theta }}_{n}$ is the local off-grid offset, and

(7) $$b\left({\tilde{\theta }}_{n}\right)={\left.\frac{\partial a(\theta )}{\partial \theta }\right|}_{\theta ={\tilde{\theta }}_{n}}$$

is the derivative of the steering vector with respect to the angular variable.

**Figure 1:**
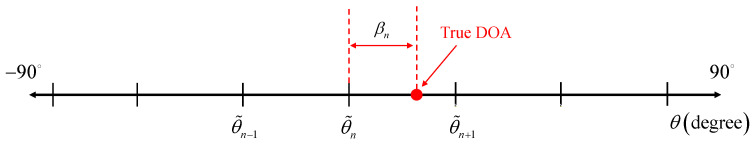
Illustration of the first-order off-grid DOA model.

Figure 1 illustrates the first-order off-grid modeling principle. The off-grid offset $\beta_{n}$ locally corrects the steering vector around the nearest grid direction, thereby reducing angular discretization mismatch.

The grid dictionary and derivative dictionary are defined as

(8) $${\left[A_{g}\right]}_{m,n}=e^{-j2\pi p_{m}\;\sin\;{\tilde{\theta }}_{n}}$$

(9) $${\left[B_{g}\right]}_{m,n}=-j2\pi p_{m}\;\cos\;{\tilde{\theta }}_{n}\;e^{-j2\pi p_{m}\;\sin\;{\tilde{\theta }}_{n}}$$

where $A_{g}\in C^{M\times N}$ is the grid dictionary and $B_{g}\in C^{M\times N}$ is the derivative dictionary.

The effective first-order off-grid dictionary is then written as

(10) $$\Phi \left(\beta \right)=A_{g}+B_{g}\;\mathrm{diag}\left(\beta \right)$$

where $\beta ={\left[\beta_{1},\beta_{2},\ldots ,\beta_{N}\right]}^{T}$ is the real-valued off-grid offset vector. To keep the first-order approximation valid, each offset is bounded by

(11) $$|\beta_{n}|\;\leq \frac{\Delta }{2}$$

where Δ is the angular grid interval.

Using the stacked observation matrix, the first-order off-grid sparse model becomes

(12) X=Φ(β)W+E

where $W\in C^{N\times L}$ is the row-sparse source coefficient matrix and $E\in C^{M\times L}$ is the effective model error matrix. Unlike the physical noise matrix N in the original array model, E includes both observation noise and residual modeling error caused by angular discretization and first-order approximation.

## 3. Proposed Method

### 3.1. Overall Framework

The proposed method follows the first-order MMV off-grid Bayesian model in Section 2. Given X, $A_{g}$, $B_{g}$, and the assumed source number *K*, it alternately updates the sparse relevance vector α, the scalar effective error precision $\alpha_{0}$, and the off-grid offset vector β. In the following derivations, Φ denotes the current effective dictionary Φ(β).

At each iteration, MMV-SBL posterior inference is performed first. Then, CGBS-MAP, PCG-DNU, and TR-CRN update α, $\alpha_{0}$, and β, respectively. After the stopping criterion is satisfied, the final DOAs are obtained from the dominant support and the refined offsets.

### 3.2. Bayesian Prior-Posterior Inference

Under the current effective dictionary Φ, the off-grid MMV observation model is written as

(13) X=ΦW+E

where $W\in C^{N\times L}$ is the row-sparse source coefficient matrix and E denotes the effective model error matrix modeled as a circularly symmetric complex Gaussian term. Let x(l) and w(l) denote the *l*th columns of X and W, respectively. For each snapshot, the likelihood is given by

(14) $$p\left(x\left(l\right)|w\left(l\right),\alpha_{0}\right)=\mathrm{CN}\left(x\left(l\right)|\Phi w\left(l\right),\alpha_{0}^{-1}I_{M}\right)$$

where $\alpha_{0}$ is the scalar precision of the effective Gaussian error. Assuming conditional independence among snapshots, the likelihood of the whole observation matrix is

(15) $$p\left(X|W,\alpha_{0}\right)=\prod_{l=1}^{L}p\left(x\left(l\right)|w\left(l\right),\alpha_{0}\right)$$

To impose common row sparsity across snapshots, each sparse coefficient vector w(l) is assigned a Gaussian prior with the shared covariance matrix:

(16) $$p\left(w\left(l\right)|\alpha \right)=\mathrm{CN}\left(w(l)|0,\Gamma \right),\;\Gamma =\mathrm{diag}\left(\alpha \right)$$

where $\alpha ={\left[\alpha_{1},\alpha_{2},\ldots ,\alpha_{N}\right]}^{T}$ is the variance-type relevance hyperparameter vector. The same α is shared by all snapshots, so that the rows of W are encouraged to have a common sparse support. The joint prior is

(17) $$p\left(W|\alpha \right)=\prod_{l=1}^{L}p\left(w\left(l\right)|\alpha \right)$$

According to Bayes’ rule, the posterior distribution is

(18) $$p\left(W|X,\alpha ,\alpha_{0}\right)\propto p\left(X|W,\alpha_{0}\right)p\left(W|\alpha \right)$$

Since both the likelihood and the prior are complex Gaussian, the posterior distribution is also complex Gaussian:

(19) $$p\left(W|X,\alpha ,\alpha_{0}\right)=\prod_{l=1}^{L}\mathrm{CN}\left(w(l)|\mu (l),\Sigma \right)$$

The posterior covariance matrix shared by all snapshots and the posterior mean of the *l*th snapshot are given by

(20) $$\Sigma ={\left(\alpha_{0}\Phi^{H}\Phi +\Gamma^{-1}\right)}^{-1}$$

(21) $$\mu \left(l\right)=\alpha_{0}\Sigma \Phi^{H}x\left(l\right)$$

For computational convenience, the equivalent form based on the matrix inversion lemma is used:

(22) $$\Sigma =\Gamma -\Gamma \Phi^{H}C^{-1}\Phi \Gamma$$

where

(23) $$C=\Phi \Gamma \Phi^{H}+\alpha_{0}^{-1}I_{M}$$

The posterior mean matrix of all snapshots is then obtained as

(24) $$M=\Gamma \Phi^{H}C^{-1}X$$

The *n*th row $m_{n}$ of M contains the posterior coefficient estimates of the *n*th candidate direction across all snapshots.

For sparse relevance learning, let

(25) $$s_{n}={\left[\Sigma \right]}_{n,n}$$

denote the posterior variance of the *n*th candidate direction. The posterior second-moment statistic is defined as

(26) $$q_{n}=\frac{1}{L}{∥m_{n}∥}_{2}^{2}+s_{n}$$

Here, $q_{n}$ combines the average posterior coefficient energy and the posterior uncertainty of the *n*th candidate direction.

In conventional SBL, the relevance parameter is usually updated directly as $\alpha_{n}=q_{n}$. However, in off-grid DOA estimation, finite snapshots, effective model error, and residual basis mismatch may produce weak and unreliable activations around the true support. Therefore, the proposed method introduces a confidence-guided MAP-type shrinkage update for $\alpha_{n}$, as described in the next subsection.

### 3.3. Confidence-Guided MAP-Type Shrinkage Relevance Learning

To improve posterior spectral concentration, CGBS-MAP introduces a reliability-controlled shrinkage mechanism into the relevance update. This design aims to reduce weak and unreliable activations while retaining dominant posterior components. Compared with direct moment updating, the shrinkage form provides a smoother way to suppress low-confidence components. It also avoids introducing a hard support threshold before the posterior relevance profile becomes sufficiently concentrated. The following exponential form is introduced as an empirical-Bayes-inspired regularization term for $\alpha_{n}$:

(27) $$p\left(\alpha_{n}|\tau_{n}\right)\propto \exp\left(-\tau_{n}\alpha_{n}\right),\;\alpha_{n}>0$$

Here, $\tau_{n}$ is not treated as a fixed prior parameter independent of the data. Instead, it is constructed adaptively from posterior reliability information and is used as a data-adaptive regularization coefficient. The resulting update is a reliability-guided regularized MAP-type shrinkage rule and does not correspond to a fully hierarchical Bayesian prior model.

The corresponding MAP-type relevance update is formulated as

(28) $$\alpha_{n}^{\mathrm{MAP}}=\arg\min_{\alpha_{n}>0}\left(\log\alpha_{n}+\frac{q_{n}}{\alpha_{n}}+\tau_{n}\alpha_{n}\right)$$

Solving the stationary condition gives the unique positive solution

(29) $$\alpha_{n}^{\mathrm{MAP}}=\frac{2q_{n}}{1+\sqrt{1+4\tau_{n}q_{n}}}$$

When $\tau_{n}=0$, the update reduces to the ordinary SBL moment update $\alpha_{n}=q_{n}$. When $\tau_{n}>0$, it provides a non-expansive shrinkage of $q_{n}$, thereby suppressing weak posterior components without hard thresholding.

The shrinkage strength is controlled by a posterior reliability score. For the scalar effective error precision considered in this paper, the effective error variance estimate is

(30) $${\hat{\sigma }}^{2}=\frac{1}{\alpha_{0}}$$

The global confidence factor and local dominance factor are defined as

(31) $$c_{n}=\frac{q_{n}}{q_{n}+\kappa_{\sigma }{\hat{\sigma }}^{2}+\varepsilon }$$

and

(32) $$d_{n}=\frac{q_{n}}{\max_{i\in N_{n}}q_{i}+\varepsilon }$$

Here, $N_{n}$ denotes a fixed local neighborhood including the *n*th grid point, ε avoids numerical division by zero, and $\kappa_{\sigma }$ is a scale coefficient. In the experiments, $N_{n}$ includes the *n*th grid point and its available immediate neighboring grid points. The factor $c_{n}$ measures the error-relative significance of $q_{n}$, whereas $d_{n}$ measures its local dominance.

The reliability score is constructed as

(33) $$z_{n}=c_{n}^{\gamma_{c}}d_{n}^{\gamma_{d}}$$

where $\gamma_{c}$ and $\gamma_{d}$ are fixed shaping constants. The adaptive shrinkage rate is then assigned by

(34) $$\tau_{n}=\mathrm{clip}\left(\frac{\lambda }{z_{n}+\varepsilon },\tau_{\min},\tau_{\max}\right)$$

where clip(x,a,b)=min{max(x,a),b}, λ>0 controls the overall shrinkage strength, and $\tau_{\min}$ and $\tau_{\max}$ are fixed bounds.The clipping operation keeps $\tau_{n}$ within a prescribed range and avoids either negligible or excessive shrinkage. A larger $z_{n}$ leads to weaker shrinkage, while a smaller $z_{n}$ leads to stronger suppression of unreliable components.

Since $\tau_{n}\geq 0$, the following non-expansive property holds:

(35) $$0<\alpha_{n}^{\mathrm{MAP}}\leq q_{n}$$

Thus, CGBS-MAP does not amplify the ordinary posterior moment update, but selectively shrinks unreliable components according to their confidence and local dominance.

For numerical stability, a damped relevance update is used:

(36) $$\alpha_{n}^{\left(t+1\right)}=\eta_{\alpha }\alpha_{n}^{\mathrm{MAP}}+\left(1-\eta_{\alpha }\right)\alpha_{n}^{\left(t\right)},\;0<\eta_{\alpha }\leq 1$$

where $\eta_{\alpha }$ is the damping factor for relevance updating. Therefore, CGBS-MAP preserves the closed-form SBL relevance update structure while introducing reliability-aware shrinkage to improve posterior spectral concentration.

**Figure 2:**
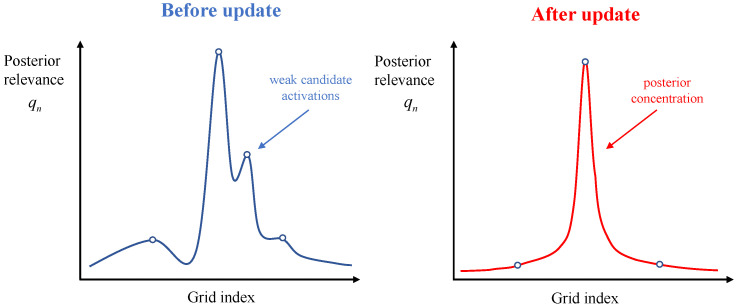
Illustration of the CGBS-MAP relevance update.

Figure 2 illustrates the reliability-controlled transformation from the posterior statistic $q_{n}$ to the MAP-type shrinkage update, where reliable components are retained and weak non-dominant activations are suppressed.

### 3.4. Posterior-Concentration-Guided Damped Noise Update

The scalar effective error precision is updated from the posterior expected reconstruction error. Under the current effective dictionary Φ, define the posterior-mean residual matrix as

(37) R=X−ΦM

For the *m*th observation dimension, the snapshot-averaged posterior residual energy is

(38) $$E_{m}^{2}=\frac{1}{L}{∥r_{m}∥}_{2}^{2}+{\left[\Phi \Sigma \Phi^{H}\right]}_{m,m}$$

where $r_{m}$ is the *m*th row of R. The first term is the posterior-mean residual energy, and the second term accounts for posterior uncertainty.

The use of $a_{0}+M$ in the numerator is due to the snapshot-averaged normalization in (38). Specifically, if the posterior expected residual energy is written in its unnormalized form, the effective residual contribution is

(39) $$\sum_{l=1}^{L}E\left[{∥x\left(l\right)-\Phi w\left(l\right)∥}_{2}^{2}\right]={∥R∥}_{F}^{2}+L\;\mathrm{tr}\;\left(\Phi \Sigma \Phi^{H}\right)$$

Dividing this expression by *L* gives

(40) $$\frac{1}{L}{∥R∥}_{F}^{2}+\mathrm{tr}\;\left(\Phi \Sigma \Phi^{H}\right)=\sum_{m=1}^{M}E_{m}^{2}$$

Thus, $\sum_{m=1}^{M}E_{m}^{2}$ represents a sensor-domain sum of snapshot-averaged posterior residual energies. Under this normalized formulation, the effective number of averaged residual terms is *M*. If the unnormalized likelihood form were used directly, an equivalent expression would contain an ML-dependent numerator and the corresponding unnormalized residual energy in the denominator.

A normalized residual-based estimate of the scalar effective error precision is then written as

(41) $$\alpha_{0,\mathrm{raw}}=\frac{a_{0}+M}{b_{0}+\sum_{m=1}^{M}E_{m}^{2}}$$

where $a_{0}$ and $b_{0}$ are small positive hyperparameters. Since direct assignment may be unstable when the support is still diffuse, a damped precision update is used:

(42) $$\alpha_{0}^{\left(t+1\right)}=\left(1-\eta_{0}^{\left(t\right)}\right)\alpha_{0}^{\left(t\right)}+\eta_{0}^{\left(t\right)}\alpha_{0,\mathrm{raw}}$$

The relaxation factor is determined by the concentration of the current relevance profile. Let $S_{K}$ denote the indices of the *K* largest entries of α. The concentration index is defined as

(43) $$\kappa_{\alpha }=\frac{\sum_{n\in S_{K}}\alpha_{n}}{\sum_{n=1}^{N}\alpha_{n}+\varepsilon },\;{\bar{\kappa }}_{\alpha }=\mathrm{clip}\left(\frac{\kappa_{\alpha }-K/N}{1-K/N+\varepsilon },0,1\right)$$

where ${\bar{\kappa }}_{\alpha }\in \left[0,1\right]$ is the normalized concentration score. The damping factor is set as

(44) $$\eta_{0}^{\left(t\right)}=\eta_{\min}+\left(\eta_{\max}-\eta_{\min}\right){\bar{\kappa }}_{\alpha },\;0\leq \eta_{\min}\leq \eta_{\max}\leq 1$$

Here, $\eta_{\min}$ and $\eta_{\max}$ denote the lower and upper bounds of the precision-update relaxation factor.

**Figure 3:**
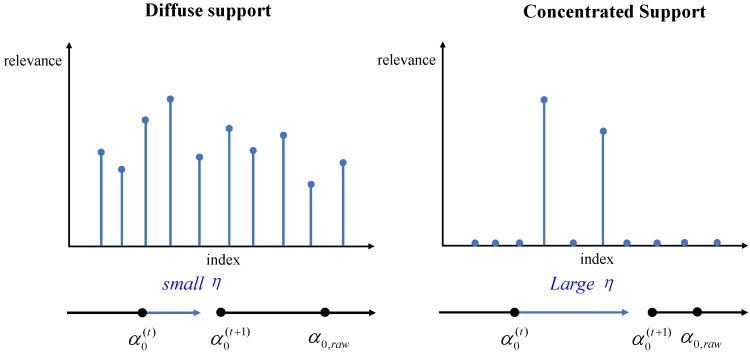
Illustration of the PCG-DNU effect. A diffuse relevance profile leads to a small relaxation factor and a conservative precision update, whereas a concentrated profile leads to a larger relaxation factor and a faster approach to the raw empirical-Bayes target.

As shown in Figure 3, PCG-DNU updates the precision conservatively when the relevance profile is diffuse and more rapidly when the support becomes concentrated.

Since (42) is a convex combination of $\alpha_{0}^{\left(t\right)}$ and $\alpha_{0,\mathrm{raw}}$, the updated precision remains bounded between them. When $\alpha_{0}^{\left(t\right)}=\alpha_{0,\mathrm{raw}}$, the update also satisfies $\alpha_{0}^{\left(t+1\right)}=\alpha_{0}^{\left(t\right)}$. Therefore, PCG-DNU stabilizes effective error precision learning while preserving the residual-based fixed point.

### 3.5. Trust-Region Cubic-Regularized Off-Grid Refinement

After sparse relevance learning, off-grid refinement is performed on the active support to reduce residual basis mismatch. Let the active support be I, whose size is $K_{a}\geq K$, selected from the $K_{a}$ largest entries of α. For fixed posterior mean M and covariance Σ, the offset correction is based on the posterior expected reconstruction error:

(45) $$L_{\beta }\left(\beta \right)={∥X-\Phi (\beta )M∥}_{F}^{2}+L\;\mathrm{tr}\left[\Phi \left(\beta \right)\Sigma \Phi^{H}\left(\beta \right)\right]$$

For clarity, the derivation of the active-support quadratic form is given below. Let the active offset vector be $u=\beta_{I}$ and write the dictionary perturbation on the active support as

(46) $$\Phi \left(u\right)=A_{g}+\sum_{i\in I}u_{i}G_{i},\;G_{i}=b_{i}e_{i}^{T}$$

where $b_{i}$ is the *i*th column of $B_{g}$ and $e_{i}$ is the *i*th canonical basis vector. Then

(47) $$\Phi \left(u\right)M=A_{g}M+\sum_{i\in I}u_{i}D_{i},\;D_{i}=b_{i}m_{i}$$

where $m_{i}$ is the *i*th row of M. Defining $R_{A}=X-A_{g}M$, the residual term becomes

(48) $${∥R_{A}-\sum_{i\in I}u_{i}D_{i}∥}_{F}^{2}$$

Because the offset variables $u_{i}$ are real-valued while $D_{i}$ and $G_{i}$ are complex-valued, the real part of the corresponding complex inner products determines the real-valued gradient and Hessian terms. Expanding the residual term and the trace term in (45), and removing constants independent of u, yields a real quadratic function of u.

Let $u=\beta_{I}$. Since the offsets are angular corrections, u is real-valued. Under the first-order off-grid model, (45) leads to

(49) $$\min_{u}\frac{1}{2}u^{T}Pu-v^{T}u\;\left|u_{i}\right|\leq \frac{\Delta }{2},\;i\in I$$

where Δ is the angular grid interval, $P\in R^{K_{a}\times K_{a}}$ is the real-valued quadratic coefficient matrix, and $v\in R^{K_{a}}$ is the real-valued linear coefficient vector. For i,j∈I,

(50) Pij=2RetrDiHDj+LtrGiΣGjH,vi=2RetrDiHRA−LtrGiΣAgH

Here, $R_{A}=X-A_{g}M$, $D_{i}=b_{i}m_{i}$, and $G_{i}=b_{i}e_{i}^{T}$. The operator Re{·} ensures that P and v are real-valued quantities associated with the real offset vector u. This treatment is equivalent to applying real-valued calculus to the real scalar cost function $L_{\beta }$.

**Figure 4:**
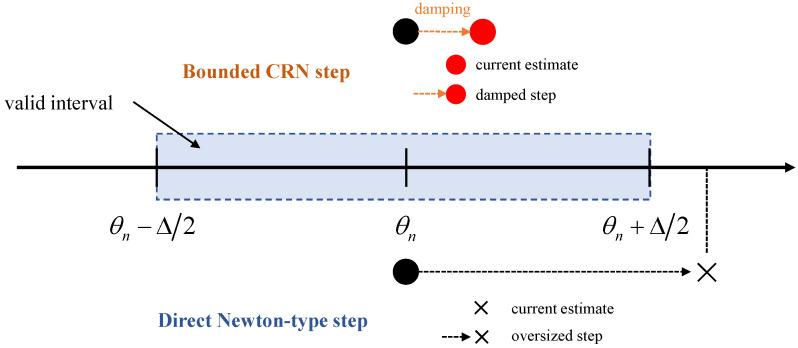
Illustration of the trust-region cubic-regularized off-grid refinement.

A direct Newton-type update may be unstable when P is ill-conditioned or when the offset step leaves the local valid range. Therefore, TR-CRN introduces cubic damping and trust-region constraints to stabilize active-support refinement. Figure 4 depicts the procedure of the trust-region cubic-regularized off-grid refinement.

Let $P_{\mathrm{reg}}=P+\delta I$, where δ>0 is a small diagonal loading factor, and define

(51) $$g_{i}={\left(P_{\mathrm{reg}}u-v\right)}_{i},\;H_{i}={\left(P_{\mathrm{reg}}\right)}_{ii}$$

TR-CRN uses the one-dimensional cubic-regularized model

(52) $$m_{i}\left(d\right)=g_{i}d+\frac{1}{2}H_{i}d^{2}+\frac{\sigma_{c}}{3}{\left|d\right|}^{3}$$

where $\sigma_{c}>0$ is the cubic regularization coefficient. The stationary condition gives

(53) $$d_{i}=-\frac{g_{i}}{H_{i}+\lambda_{i}},\;\lambda_{i}=\frac{-H_{i}+\sqrt{H_{i}^{2}+4\sigma_{c}\left|g_{i}\right|}}{2}$$

The term $\lambda_{i}\geq 0$ increases the effective curvature and suppresses oversized Newton steps.

To keep the first-order approximation valid, both the offset and update step are bounded:

(54) $$|\beta_{i}|\leq \frac{\Delta }{2},\;\left|d_{i}\right|\leq \rho \Delta ,\;0<\rho \leq \frac{1}{2}$$

The update is accepted only when $L_{\beta }$ does not increase under fixed posterior statistics. Thus, TR-CRN provides bounded active-support off-grid offset correction rather than new support discovery.

### 3.6. DOA Extraction and Overall Iterative Procedure

After the stopping criterion is satisfied, the final DOA estimates are obtained from the dominant sparse support and the corresponding off-grid offsets. Since α is a variance-type relevance vector, its large entries indicate candidate grid points with stronger posterior source evidence. Let $I_{K}=\left\{i_{1},i_{2},\ldots ,i_{K}\right\}$ denote the indices of the *K* largest entries of the final relevance vector α. The continuous-angle estimate is then obtained by adding the refined offset to the corresponding grid direction:

(55) $${\hat{\theta }}_{k}={\tilde{\theta }}_{i_{k}}+\beta_{i_{k}},\;k=1,2,\ldots ,K$$

Here, ${\tilde{\theta }}_{i_{k}}$ is the selected grid point and $\beta_{i_{k}}$ is the refined off-grid offset. The estimate ${\hat{\theta }}_{k}$ and the corresponding estimation error are measured in degrees. The estimated DOAs are sorted in ascending angular order for performance evaluation.

The iterative procedure stops when the relative change of the relevance vector becomes smaller than a prescribed tolerance, or when the maximum number of iterations is reached. The complete iterative procedure is summarized in Algorithm 1.
**Algorithm 1** Overall procedure of the proposed reliability-guided off-grid SBL method**Input:** Observation matrix X, grid dictionary $A_{g}$, derivative dictionary $B_{g}$, assumed source number *K*, active support size $K_{a}$, maximum iteration number $T_{\max}$, and convergence tolerance $\epsilon_{\mathrm{tol}}$.**Output:** Estimated DOAs ${\left\{{\hat{\theta }}_{k}\right\}}_{k=1}^{K}$ in egrees.1. Initialize $\alpha^{\left(0\right)}$, $\alpha_{0}^{\left(0\right)}$, $\beta^{\left(0\right)}=0$, and set t=0.2. Construct the effective dictionary $\Phi^{\left(t\right)}=A_{g}+B_{g}\mathrm{diag}\left(\beta^{\left(t\right)}\right)$.3. Perform MMV-SBL posterior inference with $\Phi^{\left(t\right)}$ to obtain $M^{\left(t\right)}$, $\Sigma^{\left(t\right)}$, and $q_{n}^{\left(t\right)}$.4. Update the sparse relevance vector $\alpha^{\left(t+1\right)}$ using CGBS-MAP.5. Update the scalar effective error precision $\alpha_{0}^{\left(t+1\right)}$ using PCG-DNU.6. Select the active support from the $K_{a}$ largest entries of $\alpha^{\left(t+1\right)}$, where $K_{a}\geq K$.7. Refine the active-support off-grid offsets using TR-CRN under the trust-region constraint and update $\beta^{\left(t+1\right)}$ accordingly.8. If the convergence criterion is satisfied or $t\geq T_{\max}$, stop; otherwise set t=t+1 and return to Step 2.9. Select the final support $I_{K}$ from the *K* largest entries of α and extract final DOAs by ${\hat{\theta }}_{k}={\tilde{\theta }}_{i_{k}}+\beta_{i_{k}}$.

The proposed framework combines CGBS-MAP, PCG-DNU, and TR-CRN within a unified first-order MMV Bayesian inference procedure. These modules respectively improve relevance concentration, stabilize effective error precision learning, and provide bounded active-support off-grid offset correction. The final DOA estimates are obtained from the learned support and refined offsets.

It should be noted that the complete alternating procedure is not claimed to provide a strict monotonic decrease in a single global objective, because the relevance update and precision update include reliability-guided shrinkage and damping. The acceptance rule in TR-CRN only ensures that the offset-related posterior expected reconstruction error does not increase under fixed posterior statistics. Therefore, the proposed method is regarded as a stabilized iterative inference scheme. Accordingly, the experiments use the relative change of the relevance vector and the maximum iteration number as the stopping criteria.

### 3.7. Hyperparameter Determination and Reproducibility Settings

The key hyperparameters of the proposed method are $\gamma_{c}$, $\gamma_{d}$, $\tau_{\min}$, $\tau_{\max}$, and ρ. These parameters control the reliability shaping, adaptive shrinkage range, and trust-region offset refinement of the proposed method. To improve reproducibility and avoid case-specific tuning, the same default values are used in all simulation experiments unless otherwise stated. Their influence is further examined by the sensitivity analysis in Section 4.5.

For the CGBS-MAP module, $\gamma_{c}$ and $\gamma_{d}$ control the nonlinear shaping of the posterior confidence factor and the local dominance factor in (33), respectively. The bounds $\tau_{\min}$ and $\tau_{\max}$ in (34) restrict the adaptive shrinkage rate and prevent it from becoming either too weak or excessively strong. For the TR-CRN module, ρ in (54) controls the maximum offset update in one iteration. Since the first-order off-grid model is valid only in a local angular region, ρ is chosen to be smaller than 1/2. The key hyperparameters for the experiments are presented in Table 1.

The values $\gamma_{c}=0.8$ and $\gamma_{d}=1.2$ provide moderate shaping of the confidence and local dominance factors. The bounds $\tau_{\min}=0.25$ and $\tau_{\max}=4.0$ restrict the adaptive shrinkage range so that unreliable components are suppressed without over-shrinking dominant components. The trust-region ratio ρ=0.10 gives a conservative bounded offset step and helps keep the first-order approximation within its local valid region. The remaining implementation constants are fixed as $\lambda =10^{-3}$, $\kappa_{\sigma }=1$, $\eta_{\alpha }=0.8$, $\eta_{\min}=0.1$, $\eta_{\max}=0.8$, $\delta =10^{-6}$, $\sigma_{c}=10^{-2}$, $a_{0}=b_{0}=10^{-6}$, and $\varepsilon =10^{-12}$. These constants are used for numerical scaling, damping, diagonal loading, cubic regularization, precision-update initialization, and numerical safeguards. They are kept unchanged in all experiments and are not tuned for individual cases.

### 3.8. Computational Complexity Analysis

Let *T* denote the average number of outer iterations. In each iteration, the main computational cost comes from MMV-SBL posterior inference. Constructing the data covariance matrix $C=\Phi \Gamma \Phi^{H}+\alpha_{0}^{-1}I_{M}$ requires approximately $O(M^{2}N)$ operations. Factorizing the M×M matrix C, or solving the corresponding linear systems, requires approximately $O(M^{3})$ operations. Once the factorization of C is available, computing the posterior mean matrix $M=\Gamma \Phi^{H}C^{-1}X$ requires approximately $O(M^{2}L+MNL)$ operations. Computing the diagonal posterior variances required by $q_{n}$ requires approximately O(MN) operations when only the diagonal entries of Σ are evaluated.

The CGBS-MAP relevance update mainly consists of element-wise operations over the *N* grid points and therefore introduces approximately O(N) additional cost when the posterior statistics are already available. The PCG-DNU update uses the posterior residual energy and the relevance concentration score. Apart from residual evaluation, its additional damping operation is scalar and has low computational cost. For TR-CRN, the active support size is $K_{a}$. Forming the active-support quadratic terms requires approximately $O(K_{a}^{2}ML+K_{a}^{2}M)$ operations, and the coordinate-wise cubic-regularized update requires $O(K_{a})$ operations. Since $K_{a}\ll N$ in the experiments, the TR-CRN overhead is limited to the selected active support rather than the full angular grid.

Combining the dominant terms, the approximate per-run complexity of the proposed method is

(56) $$O\left(T\left(M^{2}N+M^{3}+M^{2}L+MNL+K_{a}^{2}ML\right)\right).$$

Thus, the proposed method has a higher computational burden than greedy or root-based baselines, mainly because it retains iterative MMV Bayesian posterior inference and additionally performs reliability-guided stabilization and active-support off-grid refinement.

## 4. Simulation Results and Discussion

This section evaluates the proposed method through eight controlled experiments: off-grid deviation evaluation, SNR sweep, snapshot sweep, hyperparameter sensitivity analysis, source-separation evaluation, random-angle generalization, ablation study, and runtime comparison. These experiments examine off-grid compensation accuracy, noise-level sensitivity, multisnapshot performance, parameter robustness, scenario generalization, module contribution, and computational burden under a consistent simulation protocol.

### 4.1. Performance Metrics and Experimental Settings

The main experimental settings are summarized in Table 2. For each experimental setting, all compared methods are evaluated under the same Monte Carlo protocol.

Unless otherwise specified, the compared baselines include MUSIC, Root-MUSIC, ESPRIT, OMP, OGSBL, RootSBL, and OGFVBI, while several supplementary experiments report a representative subset for clarity. The SNR is defined as

(57) $$\mathrm{SNR}=10\log_{10}\frac{{∥A\left(\theta \right)S∥}_{F}^{2}}{{∥N∥}_{F}^{2}}.$$

For each target SNR, the complex Gaussian noise variance is adjusted according to (57). Unless otherwise stated, the sources have equal powers and mutually independent waveforms.

RMSE and success rate are used to evaluate estimation accuracy and localization reliability. Since the estimated DOAs may not be ordered consistently with the true DOAs, the estimates are first matched to the true directions by the minimum total angular error in each Monte Carlo trial. All DOA estimates and angular errors are measured in degrees. The RMSE is defined as

(58) $$\mathrm{RMSE}=\sqrt{\frac{1}{KP}\sum_{r=1}^{P}\sum_{k=1}^{K}{\left({\hat{\theta }}_{k}^{\left(r\right)}-\theta_{k}\right)}^{2}}$$

where *K* is the number of sources, *P* is the number of Monte Carlo trials, $\theta_{k}$ is the true DOA, and ${\hat{\theta }}_{k}^{\left(r\right)}$ is the matched estimate in the *r*th trial.

A trial is regarded as successful only when all matched DOA errors are smaller than a prescribed tolerance τ. The success rate is defined as

(59) $$\mathrm{Success}\;\mathrm{Rate}=\frac{1}{P}\sum_{r=1}^{P}I\;\left(\max_{1\leq k\leq K}\left|{\hat{\theta }}_{k}^{\left(r\right)}-\theta_{k}\right|<\tau \right)\times 100\%$$

where I(·) is the indicator function. Unless otherwise stated, $\tau =0.5^{{}^{\circ}}$, which is stricter than the $1^{{}^{\circ}}$ grid interval. For the single-source off-grid deviation experiment, the absolute error $e_{\theta }=\left|\hat{\theta }-\theta_{\mathrm{true}}\right|$ is reported in degrees.

### 4.2. Off-Grid Deviation Evaluation

To directly evaluate the off-grid compensation capability of different methods, a single-source off-grid deviation experiment is conducted. The same M=8 half-wavelength ULA and $1^{{}^{\circ}}$ angular grid are used. The nearest grid point is fixed at $\theta_{\mathrm{grid}}=0^{{}^{\circ}}$, and the true DOA is moved away from this grid point by different fractions of the grid interval. The normalized off-grid deviation ratio is defined as

(60) $$\rho_{\mathrm{off}}=\frac{\left|\theta_{\mathrm{true}}-\theta_{\mathrm{grid}}\right|}{\Delta_{g}}$$

where $\Delta_{g}=1^{{}^{\circ}}$ is the grid interval. In this experiment, $\rho_{\mathrm{off}}$ is set to 0, 0.1, 0.2, 0.3, and 0.4, corresponding to true DOAs of $0^{{}^{\circ}}$, $0.1^{{}^{\circ}}$, $0.2^{{}^{\circ}}$, $0.3^{{}^{\circ}}$, and $0.4^{{}^{\circ}}$, respectively. The number of snapshots is fixed at L=100, the SNR is fixed at 5 dB, and K=1 is used only for this deviation test.

For each value of $\rho_{\mathrm{off}}$, Table 3 reports the absolute estimation error

(61) $$e_{\theta }=\left|\hat{\theta }-\theta_{\mathrm{true}}\right|$$

The left column denotes the normalized off-grid deviation ratio, and the remaining columns give the estimation errors of different algorithms in degrees.

Table 3 shows that the proposed method achieves the lowest average error among the compared methods in this illustrative single-source setting, while RootSBL also provides very close average performance. It should also be noted that the proposed method is not uniformly best at every individual deviation ratio; RootSBL and OGFVBI obtain smaller errors at some points. Therefore, this experiment is interpreted as a direct demonstration of off-grid correction behavior rather than a complete proof of general off-grid superiority. In contrast, OMP is restricted to the fixed angular grid, so its error increases as the true DOA moves away from the grid point. The subsequent source-separation and random-angle experiments further complement this single-source off-grid test by examining multi-source and randomly generated angular scenarios.

### 4.3. Performance Under Varying SNR

The SNR experiment evaluates the estimation accuracy and localization reliability of different methods under varying noise levels. Unless otherwise stated, the common settings in Table 2 are used. In this experiment, the number of snapshots is fixed at L=200, while the SNR is varied from −10 dB to 10 dB. Each point is averaged over P=100 Monte Carlo trials, and the success threshold is set to $0.5^{{}^{\circ}}$. This experiment examines whether each method can maintain accurate and reliable off-grid localization as the observation noise level changes.

**Figure 5:**
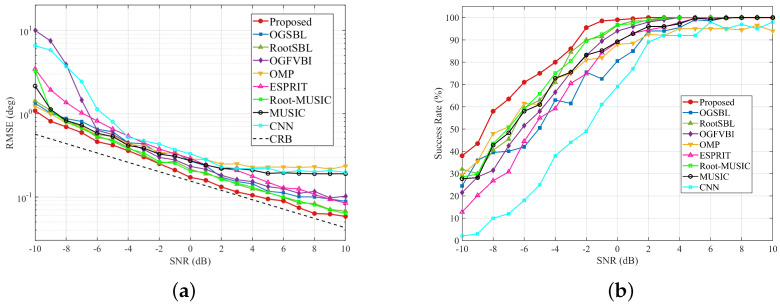
Performance comparison under varying SNR. (**a**) RMSE versus SNR. (**b**) Success rate versus SNR. The number of snapshots is fixed at L=200, and each point is averaged over 100 Monte Carlo trials. A trial is counted as successful only when all matched DOA errors are smaller than $0.5^{{}^{\circ}}$.

Figure 5a shows that the RMSE of most methods decreases as the SNR increases. OMP exhibits an error floor because it is restricted to the fixed angular grid. OGSBL, RootSBL, and OGFVBI improve the estimation accuracy through off-grid refinement or Bayesian inference, while MUSIC, Root-MUSIC, and ESPRIT provide classical subspace-based references. The CNN baseline also shows improved accuracy as the SNR increases, but its performance is limited by the grid-based output and is less stable in the low-SNR region. The proposed method achieves lower RMSE at several SNR points, especially in the low-to-medium SNR region.

Figure 5b shows that the success rates generally increase with SNR. The CNN baseline has a relatively low success rate at low SNRs but improves significantly as the SNR increases. The proposed method obtains a higher success rate at most low-to-medium SNR points, while several methods approach high success rates as the SNR increases.

This behavior is consistent with the design of the reliability-guided inference chain: CGBS-MAP improves relevance concentration, PCG-DNU stabilizes effective error precision learning, and TR-CRN provides bounded active-support offset correction.

### 4.4. Performance Under Varying Snapshot Numbers

This experiment investigates the influence of the number of snapshots on estimation accuracy and localization reliability. The common settings in Table 2 are used, with the SNR fixed at 5 dB. The number of snapshots is varied from 50 to 500, and each point is averaged over P=100 Monte Carlo trials. This setting evaluates whether each method can maintain reliable off-grid localization under different amounts of available temporal observations.

**Figure 6:**
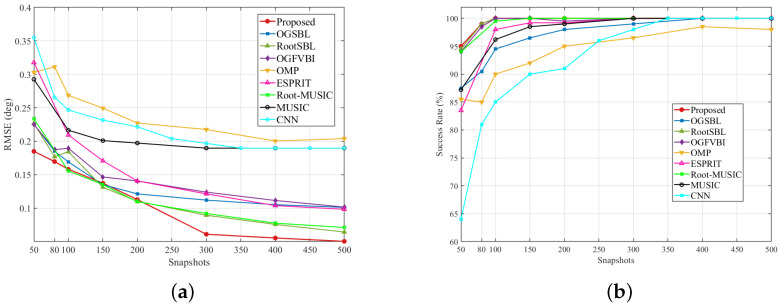
Performance comparison under varying snapshot numbers. (**a**) RMSE versus the number of snapshots. (**b**) Success rate versus the number of snapshots. The SNR is fixed at 5 dB, and each point is averaged over 100 Monte Carlo trials. The success threshold is $0.5^{{}^{\circ}}$.

Figure 6a shows that the RMSE values of most methods decrease as the number of snapshots increases, indicating that additional multisnapshot data improve estimation reliability. OMP remains limited by fixed-grid mismatch, whereas the off-grid Bayesian methods benefit from continuous-angle refinement. The CNN baseline also benefits from the increase in snapshots because a larger number of snapshots provides a more stable sample covariance matrix. The proposed method maintains low RMSE over the tested snapshot range, showing stable estimation accuracy under different data lengths.

Figure 6b presents the corresponding success-rate curves. The CNN baseline shows an increasing success rate as the number of snapshots increases, but its reliability is lower when the number of snapshots is small. The proposed method maintains high localization reliability over the tested snapshot range and remains competitive with RootSBL, Root-MUSIC, and OGFVBI. This behavior is consistent with the reliability-guided inference chain: CGBS-MAP improves support concentration, PCG-DNU stabilizes effective error precision learning, and TR-CRN refines the offsets within a bounded local region.

### 4.5. Hyperparameter Sensitivity Analysis

To examine the influence of the key hyperparameters, a one-factor-at-a-time sensitivity analysis is conducted. The basic setting is M=8, K=2, $\theta =[-15.24^{{}^{\circ}},15.12^{{}^{\circ}}]$, L=200, Gaussian noise, and P=200 Monte Carlo trials. For each test, only one parameter is varied while the other parameters are fixed at the default values in Table 1. The tested hyperparameters include $\gamma_{c}$, $\gamma_{d}$, $\tau_{\min}$, $\tau_{\max}$, and ρ. For each hyperparameter value, the SNR is varied from −10 dB to 10 dB.

**Figure 7:**
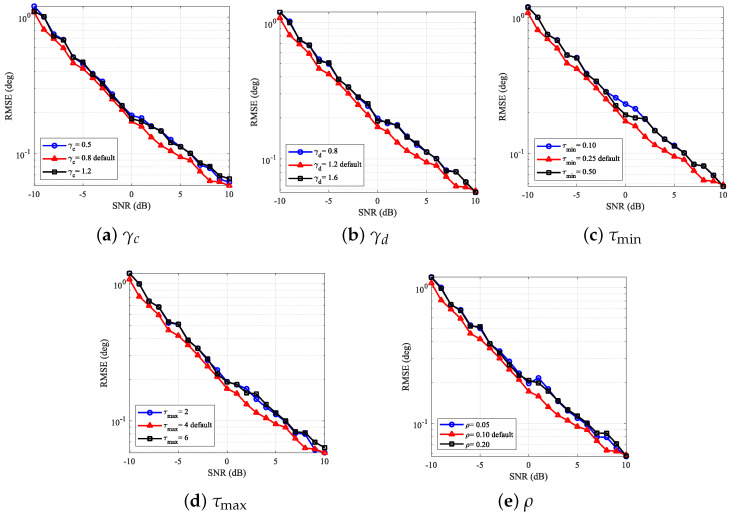
Hyperparameter sensitivity analysis of the proposed method. (**a**) $\gamma_{c}$; (**b**) $\gamma_{d}$; (**c**) $\tau_{\min}$; (**d**) $\tau_{\max}$; (**e**) ρ.

Figure 7 shows the sensitivity analysis of the key hyperparameters. In each subfigure, one parameter is varied while the remaining parameters are fixed at their default values. The RMSE curves show similar decreasing trends as the SNR increases, indicating that the proposed method does not exhibit severe sensitivity to moderate variations of a single hyperparameter. The default parameter settings generally provide stable and competitive estimation performance over the tested SNR range, without requiring case-specific tuning.

### 4.6. Performance Under Different Source Separations

To further evaluate the resolution capability of the compared methods, a two-source experiment with different angular separations is conducted. In this experiment, the SNR is fixed at 5 dB, the number of snapshots is fixed at L=200, the grid interval is $1^{{}^{\circ}}$, and each point is averaged over P=200 Monte Carlo trials.

**Figure 8:**
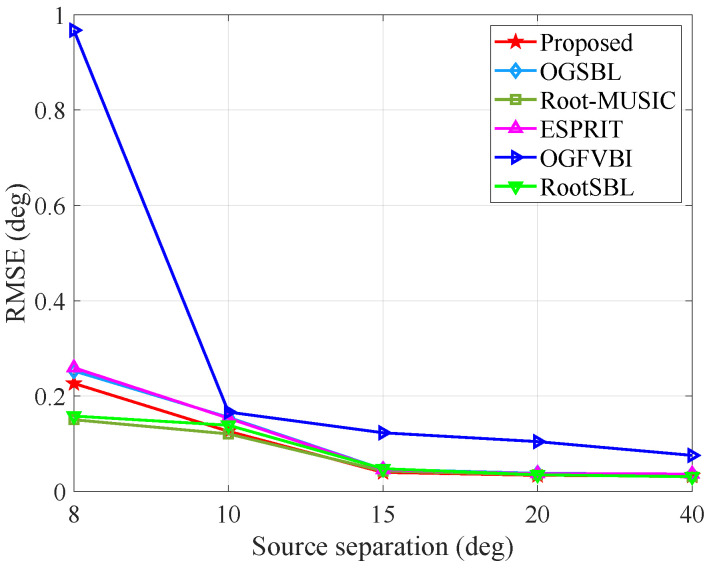
Performance comparison under different source separations. The SNR is fixed at 5 dB, the number of snapshots is fixed at L=200, and each point is averaged over 200 Monte Carlo trials.

Figure 8 shows the estimation performance under different source separations. When the two sources are closely spaced, the steering vectors become highly correlated, and the DOA estimation problem becomes more challenging. As the angular separation increases, the source distinguishability improves, and most methods achieve lower estimation errors. The proposed method maintains stable performance over the tested separation range, indicating that the reliability-guided sparse Bayesian inference and bounded off-grid refinement can effectively support multi-source localization under different angular configurations.

### 4.7. Random-Angle Generalization Experiment

To avoid evaluating the algorithms only at a fixed DOA pair, an additional random-angle experiment is conducted. In each Monte Carlo trial, the two true DOAs are independently generated from two separated angular intervals:

(62) $$\theta_{1}∼U\left(-30^{{}^{\circ}},-10^{{}^{\circ}}\right),\;\theta_{2}∼U\left(10^{{}^{\circ}},30^{{}^{\circ}}\right)$$

The SNR is fixed at 5 dB, the number of snapshots is fixed at L=200, and P=100 Monte Carlo trials are used.

Table 4 shows that the proposed method achieves a competitive RMSE of $0.1204^{{}^{\circ}}$ with a 100% success rate in the random-angle scenario. Root-MUSIC and RootSBL achieve slightly lower RMSEs because the sources are well separated and the ULA model is exactly satisfied. Nevertheless, the proposed method remains very close to these strong baselines and achieves lower RMSE than OGSBL, ESPRIT, and OGFVBI in this test. This result suggests that the proposed method is not only effective for a fixed DOA pair but also remains stable under randomly generated angular scenarios.

### 4.8. Ablation Study

To examine the contribution of each proposed inference module, an ablation study is conducted under the same basic setting as the SNR experiment. The SNR is varied from −10 dB to 10 dB, the number of snapshots is fixed at L=200, and each point is averaged over P=100 Monte Carlo trials. Four variants are compared: the complete method, denoted as Proposed-full; the method without CGBS-MAP, denoted as w/o CGBS-MAP; the method without PCG-DNU, denoted as w/o PCG-DNU; and the method without TR-CRN, denoted as w/o TR-CRN.

In the w/o CGBS-MAP variant, the sparse relevance update is replaced by the standard posterior second-moment update. In the w/o PCG-DNU variant, the scalar effective error precision is directly updated by the residual-based estimate $\alpha_{0,\mathrm{raw}}$ without concentration-guided damping. In the w/o TR-CRN variant, the cubic-regularized trust-region refinement is removed and the offset correction is performed by a regularized direct update.

**Figure 9:**
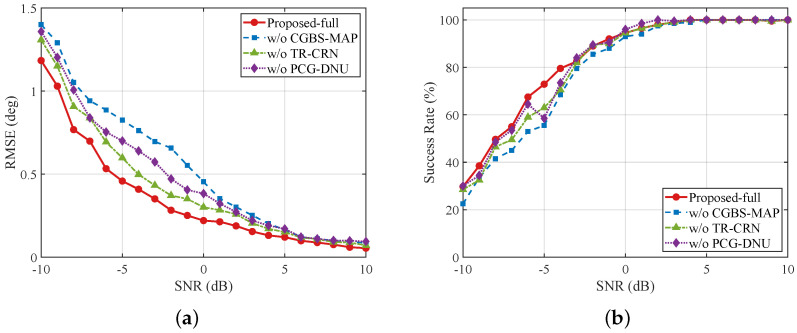
Ablation study under varying SNR. (**a**) RMSE versus SNR. (**b**) Success rate versus SNR. The complete method is compared with variants that remove CGBS-MAP, PCG-DNU, or TR-CRN. The success threshold is $0.5^{{}^{\circ}}$.

Figure 9a shows that removing any proposed module generally increases the RMSE at several SNR points. Without CGBS-MAP, weak neighboring activations are less effectively suppressed, which may affect support selection. Without PCG-DNU, the effective error precision update becomes more sensitive to uncertain support formation. Without TR-CRN, the offset correction loses the bounded cubic-regularized refinement and may become less stable. These results indicate that the three modules contribute to different stages of the inference chain.

Figure 9b shows that the complete method maintains a higher or comparable success rate at most SNR points. This means that the proposed modules improve not only the average estimation error, but also the repeatability of successful localization. Overall, the ablation results suggest that the observed performance gain is related to the combined effect of reliability-guided relevance shrinkage, concentration-guided effective error precision relaxation, and bounded off-grid refinement.

### 4.9. Computational Runtime Comparison

All simulations were performed on a computer with a 13th Gen Intel(R) Core(TM) i7-13650HX CPU and 24.0 GB RAM using MATLAB R2023b under the same framework without parallel computation. The running times are listed in Table 5.

The proposed method has the largest average runtime among the compared methods because it retains iterative MMV Bayesian posterior inference and additionally performs reliability-guided relevance shrinkage (CGBS-MAP), damped effective error precision updating (PCG-DNU), and active-support off-grid refinement (TR-CRN). Compared with other SBL-based baselines, the proposed method uses a larger iteration budget, which is reflected in the computational cost.

For the iterative SBL-based methods, the observed iteration records indicate that most algorithms reached their prescribed maximum iteration numbers under the strict convergence tolerance. Therefore, the runtime values primarily reflect the preset iteration budgets rather than natural early-stopping convergence. The main computational burden comes from the MMV-SBL posterior inference step, including covariance construction, matrix factorization, and posterior mean computation. Since TR-CRN is applied only to the selected active support and PCG-DNU is a scalar relaxation step, their additional overhead is relatively limited.

### 4.10. Discussion

The simulation results indicate that the proposed method can provide competitive and stable off-grid DOA estimation in the tested controlled scenarios by jointly enhancing support formation, effective error precision learning, and offset refinement. The off-grid deviation, SNR, and snapshot experiments show stable off-grid localization behavior under the considered mismatch, noise-level, and data-length conditions. The hyperparameter sensitivity analysis shows that the selected default parameter set provides a stable operating point, while moderate parameter variations do not lead to algorithmic breakdown. The source-separation and random-angle experiments supplement the fixed-angle tests by evaluating different angular configurations and randomly generated DOA pairs. The ablation study indicates that CGBS-MAP, PCG-DNU, and TR-CRN contribute to different stages of the inference chain, so the observed performance improvement is related to their coordinated effect rather than to a single isolated modification. In particular, improved relevance concentration provides a more reliable active support for subsequent bounded offset correction.

The improved accuracy and reliability are obtained at the cost of increased computational burden. This is mainly because the proposed method retains iterative MMV-SBL posterior inference and adds reliability-guided relevance shrinkage, damped effective error precision learning, and active-support offset refinement. However, the additional cost is mainly associated with improved inference stability and continuous-angle correction. Therefore, the proposed method is more suitable for scenarios where off-grid estimation accuracy and estimation stability are more important than minimum runtime.

## 5. Conclusions

This paper proposed a reliability-guided stabilized off-grid SBL method for multisnapshot DOA estimation within a first-order MMV Bayesian model. The method stabilizes three key inference steps: sparse relevance learning, scalar effective error precision updating, and active-support off-grid refinement. Specifically, CGBS-MAP enhances sparse relevance concentration by suppressing weak and non-dominant posterior components, PCG-DNU stabilizes effective error precision learning through concentration-guided damping, and TR-CRN provides bounded continuous-angle offset correction with cubic regularization and trust-region constraints. The proposed method is evaluated through off-grid deviation, SNR, snapshot, hyperparameter sensitivity, source-separation, random-angle, ablation, and runtime experiments. Simulation results show that the proposed method achieves competitive DOA estimation performance compared with representative baselines in the tested settings. The ablation results support the contribution of the three modules, while the runtime and theoretical complexity analyses indicate an accuracy–complexity tradeoff with increased computational burden. The current study is still limited to controlled simulation scenarios with known source number, uncorrelated sources, equal source powers, and ideal array calibration. Future work will consider extensions to more challenging scenarios, such as coherent sources, unequal source powers, unknown source number estimation, array model imperfections, and more systematic learning-based comparisons.

## Figures and Tables

**Table 1:** Default values of the key hyperparameters used in all simulation experiments.

Parameter	Default Value	Function
$\gamma_{c}$	0.8	Shape factor of posterior confidence in (33)
$\gamma_{d}$	1.2	Shape factor of local dominance in (33)
$\tau_{\min}$	0.25	Lower bound of the adaptive shrinkage rate in (34)
$\tau_{\max}$	4.0	Upper bound of the adaptive shrinkage rate in (34)
ρ	0.10	Trust-region step ratio in (54)

**Table 2:** Main experimental settings.

Item	Setting
Array geometry	Half-wavelength-spaced ULA, M=8
Angular range/grid	$\left[-90^{{}^{\circ}},90^{{}^{\circ}}\right]$, $1^{{}^{\circ}}$
Grid points	N=181
Source scenario	Two uncorrelated far-field narrowband sources
True DOAs/source number	$\left[-15.24^{{}^{\circ}},\;15.12^{{}^{\circ}}\right]$, K=2
Noise model	Circularly symmetric complex Gaussian
Monte Carlo trials	P=100
Maximum iterations/tolerance of iterative methods	100, $10^{-4}$
Active support size	$K_{a}=2K$

**Table 3:** Absolute estimation errors in degrees under different normalized off-grid deviation ratios.

Normalized Off-Grid Deviation $\rho_{\mathrm{off}}$	Proposed	OGSBL	RootSBL	OGFVBI	OMP
0.00	0.03422	0.07218	0.04170	0.01137	0.00000
0.10	0.04723	0.09947	0.03941	0.06613	0.10000
0.20	0.04286	0.09319	0.03706	0.05304	0.20000
0.30	0.04045	0.07718	0.03466	0.02446	0.30000
0.40	0.01875	0.06118	0.03224	0.09474	0.40000
Average	**0.03670 **	0.08064	0.03701	0.04995	0.20000

**Table 4:** Random-angle generalization results. The two DOAs are randomly generated from $\left[-30^{{}^{\circ}},-10^{{}^{\circ}}\right]$ and $\left[10^{{}^{\circ}},30^{{}^{\circ}}\right]$ in each Monte Carlo trial.

Method	RMSE (Deg)	Success Rate (%)
Proposed	0.1204	100.0
OGSBL	0.1657	100.0
Root-MUSIC	0.1190	100.0
ESPRIT	0.1527	100.0
OGFVBI	0.1367	99.5
RootSBL	0.1176	100.0

**Table 5:** Average runtime under the SNR sweep.

Method	Average Runtime (s)
RootSBL	0.1207
OGSBL	0.3200
OGFVBI	0.3908
Proposed	0.4152

## Data Availability

The data reported in this study are generated from numerical simulations and can be reproduced using the model parameters and experimental settings described in the manuscript. Additional simulation data are available from the corresponding author upon reasonable request.

## Abbreviations

DOA	Direction of Arrival
SBL	Sparse Bayesian Learning
MMV	Multiple Measurement Vector
MAP	Maximum a Posteriori
OMP	Orthogonal Matching Pursuit
OGFVBI	Off-Grid Fast Variational Bayesian Inference
SNR	Signal-to-Noise Ratio
RMSE	Root Mean Square Error
CGBS-MAP	Confidence-Guided MAP-Type Shrinkage Relevance Learning
PCG-DNU	Posterior-Concentration-Guided Damped Noise Update
TR-CRN	Trust-Region Cubic-Regularized Newton
CRN	Cubic-Regularized Newton
